# Supplementary material for: Effect of Light of Different Spectral Compositions on Pro/Antioxidant Status, Content of Some Pigments and Secondary Metabolites and Expression of Related Genes in Scots Pine
Source: Plants (Basel). 2023 Jul 5;12(13):2552. doi: 10.3390/plants12132552 (PMC10346266; doi:10.3390/plants12132552)
Supplement: Supplementary file 1 [file plants-12-02552-s001.zip › plants-2489015-supplementary.pdf]

Table S1. Primers for qRT-PCR analysis

	Gene Bank ID	Gene description	Gene	Primer 5′-3′	
				forward	reverse
1	B0ZQ68 (uniprot.org)	4-coumarate-CoA ligase	*4CL*	AAACTGGCGAGTCTCTACCG	ATTGTAGCGGCCGTGGATTC
2	A0A023W7L9 (uniprot.org)	cinnamate 4-hydroxylase	*CH4*	ATTCGGGAACTGGTTGCAGG	TGGCGAGATCGGGAGATGAA
3	MA_407452g0010 (congenie.org)	Phytoene synthase	*PSY*	TCAAGATGAGCTTGCACGGA	ACCGGCCATCTACTGGTTTT
4	MF395340.1 (ncbi.nlm.nih.gov)	bHLH transcription factors	*MYC*	AAAAGGCCCCGGAAAAGAGG	TTGGTACCACAGCACGAAGC
5	EF083399.1 (ncbi.nlm.nih.gov)	Jasmonate-Zim domain 1	*JAZa*	GGTGAACGTGTATGATGATATTCC	CGTTGCAGAGAATGCTTCCTC
6	GILO01669683.1 (ncbi.nlm.nih.gov)	Jasmonate-Zim domain 2	*JAZb*	ATGCTGCTCGCTGATAGTG	CGCCAGAAGTCTGAAGAGA
7	EF084624.1 (ncbi.nlm.nih.gov)	Pathogenesis-related protein 1	*PR1*	GCACTCTGGTGGTCAATACG	CACCCGAGCCTCTTGGAA
8	MZ222278.1 (ncbi.nlm.nih.gov)	Pathogenesis-related protein 5	*PR5*	CCTGCGTTGCTTTCAATAC	GCAAGTGAAGGTGCTGGT
9	BN000697.1 (uniprot.org)	leucoanthocyanidin reductase	*LAR*	TACAACGCCGAGAACAACCA	TTTCACACTGGCCTCTGCAA
10	Q8RUZ3 (uniprot.org)	phenylalanine ammonia-lyase	*PAL*	ATTGAAATGAGCGGAGCCGA	CACACAATGGAAGCCAGTGC
11	CBB44933.1 (uniprot.org)	Actin 1	*ACT1*	TTAGCAACTGGGATGACATGGA	CCTGAATGGCAACATACATAGCA

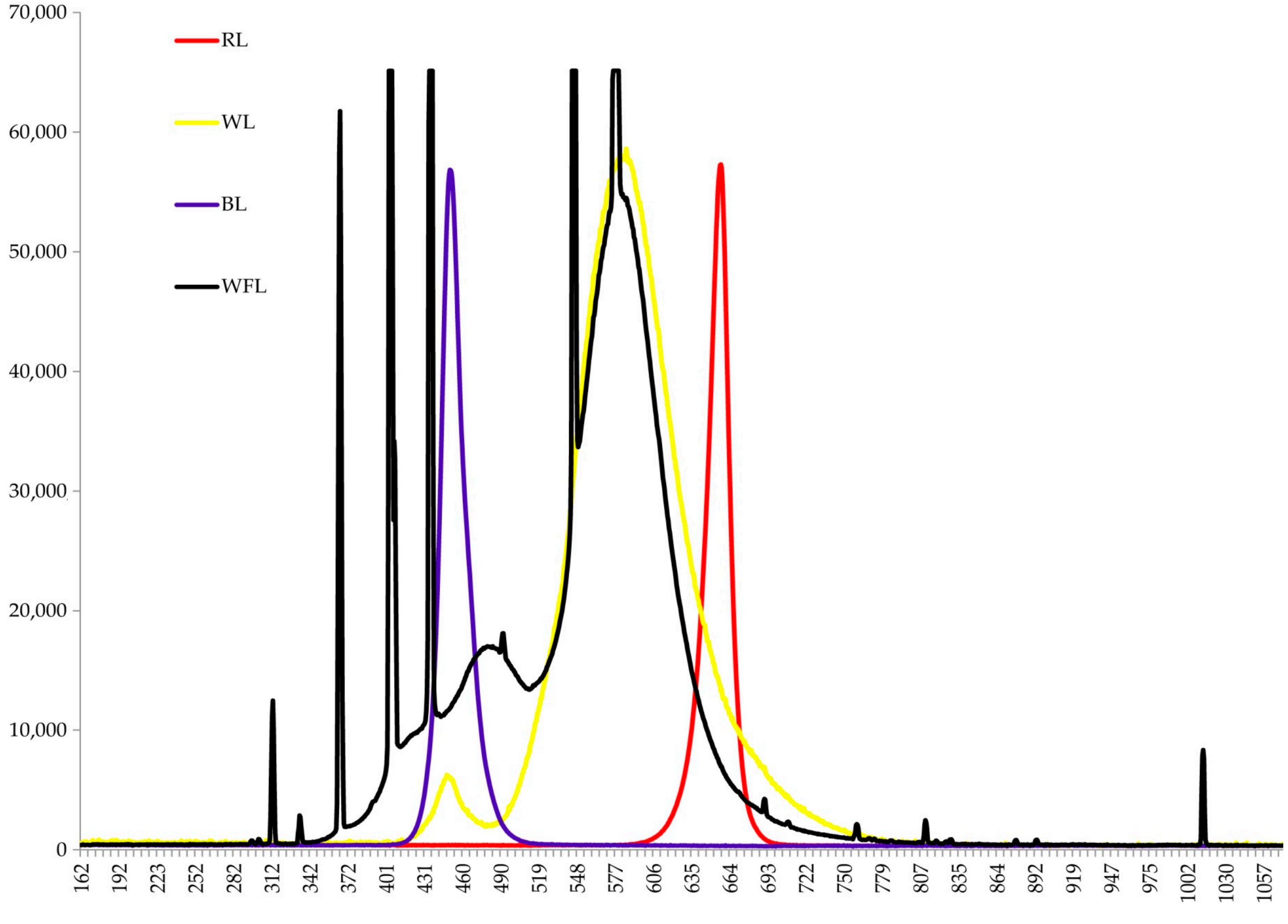

Figure S1. Emission spectra of various light sources used in the experiment.
